# Soil Biodiversity of *Eucalyptus saligna*: Insights Into Bacterial and Nematode Communities

**DOI:** 10.1111/1758-2229.70341

**Published:** 2026-04-08

**Authors:** Ebrahim Shokoohi, Peter Masoko

**Affiliations:** ^1^ Department of Biochemistry, Microbiology, and Biotechnology University of Limpopo Sovenga South Africa

**Keywords:** ecology, *Eucalyptus*, forest, microbiome, nematode, production, soil

## Abstract

Soil microorganisms and nematodes are key regulators of soil function, yet their co‐occurrence in *Eucalyptus* rhizospheres remains poorly understood. In this exploratory study, we characterised bacterial and nematode communities associated with *Eucalyptus saligna* soils in Limpopo, South Africa, using high‐throughput 16S rRNA gene sequencing and nematode surveys. Bacterial assemblages were dominated by Proteobacteria (42%), Acidobacteria (28%), Actinobacteria (12%) and Planctomycetes (9%). Eleven bacterial genera occurred across all sites, with Rhizobiales (prominence value, PV = 315,350) and Xanthobacteraceae (PV = 292,930) emerging as the most prominent taxa. Nematode surveys identified 19 genera, including plant‐parasitic such as *Meloidogyne* (PV = 5759.1) and abundant free‐living such as *Tylolaimorphus* (PV = 4150.0) and *Acrobeloides* (PV = 2900.0). Principal component analysis showed that bacterial communities were associated with soil pH, salinity and nitrogen forms, whereas nematode assemblages were associated with phosphate and sand content, together explaining 83%–90% of total variance. Network analysis indicated that all sampling sites functioned as central hubs (degree = 19–23; eigenvector centrality = 0.90–1.0), integrating nematode–bacteria associations. Key connector taxa included *Acrobeloides*, *Wilsonema* and *Aphelenchoides,* as well as bacteria such as Rhizobiales and *Acidothermus*. These findings provide a baseline framework for understanding belowground biodiversity and co‐occurrence patterns in *Eucalyptus* plantation soils.

## Graphical abstract

This exploratory study characterises bacterial and nematode assemblages in the rhizosphere of *Eucalyptus saligna* plantations in Limpopo, South Africa. High‐throughput sequencing, nematode surveys and multivariate analyses reveal co‐occurrence patterns of soil biota with soil properties, providing a baseline assessment of belowground biodiversity and associations requiring further investigation.

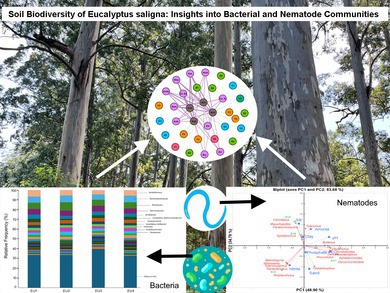

## 1 Introduction

*Eucalyptus* species rank among the most extensively cultivated broadleaf forest trees worldwide. They are currently grown in plantations across nearly 95 countries, covering more than 22.5 million hectares. These trees are well known for their rapid growth, high productivity and adaptability, particularly their resilience to poor soil conditions and their favourable stem form for timber production. Because of these traits, *Eucalyptus* has been widely adopted for diverse purposes, including large‐scale afforestation programs. Globally, it is considered one of the four major fast‐growing tree groups and together with members of the family Pinaceae, accounts for roughly 30% of plantation forests worldwide (Zhang and Wang 2021).

Over 200 species of *Eucalyptus* have been introduced into South Africa (Henderson 2009). The majority of these introductions were signified by silvicultural objectives, with the highest rate of introductions occurring between 1828 and 1940. By the end of this period, approximately 149 species had become well established in the country (Bennett 2010; Keet and Richardson 2022). While *Eucalyptus* is primarily cultivated for timber production, it also serves a wide range of purposes, including pulp and paper, charcoal, essential oil extraction, soil rehabilitation, ornamental planting and shelterbelts (Hirsch et al. 2020). Additionally, *Eucalyptus* trees are an important source of nectar and pollen, supporting beekeeping activities (Hirsch et al. 2020). Moreover, essential oils derived from *Eucalyptus* (EEOs) have demonstrated notable activity against pathogenic bacteria, fungi and viruses (Shiekh et al. 2025). Overall, these trees provide significant economic and ecological benefits, with more than 500,000 ha under cultivation in South Africa (Bennett 2011).

*Eucalyptus* trees are known to be susceptible to several plant‐parasitic nematodes, with root‐knot nematodes (*Meloidogyne* spp.) recognised as the most damaging group (Lopes Vieira et al. 2024). By contrast, the role of free‐living bacterivorous nematodes has received far less attention. In South Africa, a diverse assemblage of plant‐parasitic nematodes has been documented in association with *Eucalyptus*, including *Pratylenchus brachyurus* , *P. penetrans* , *Rotylenchus unisexus*, *Helicotylenchus dihystera*, *Scutellonema brachyurus*, *Rotylenchulus parvus*, *Meloidogyne* spp., *Criconema mutabile*, *Criconemoides parvus*, *Hemicriconemoides cocophilus*, *Mesocriconema* spp., *Ogma* spp., *Hemicycliophora typica*, *Paratrichodorus lobatus*, *Xiphinema mampara* f. *minor* and *X. xenovariabile* (Marais and Swart 2003).

A microbiome study on *Eucalyptus urograndis* in Brazil revealed that six bacterial genera—*Mycobacterium*, *Bradyrhizobium*, *Streptomyces*, *Bacillus*, *Actinospica* and *Burkholderia*—accounted for over 50% of the classified sequences (Fonseca et al. 2018). Similarly, an investigation of the *Eucalyptus pellita* microbiome in Indonesia identified *Acidothermus* as the most dominant genus, followed by *Candidatus Solibacter*, *Acidibacter* and *Variibacter*, while *Labrys* and *Geobacter* were among the least represented. In South Africa, several bacterial pathogens associated with *Eucalyptus* leaves have been documented (Rikhotso 2021). For instance, *Teratosphaeria nubilosa* has been recognised as a major factor leading to the discontinuation of *E. globulus* plantations in South Africa (Lundquist and Purnell 1987). In addition, *Pantoea agglomerans* has been reported as a pathogen affecting the aerial parts of *Eucalyptus* in South Africa. Despite these findings, no comprehensive research has yet been conducted on the microbiome associated with the rhizosphere soil of *Eucalyptus* in the country.

Although *Eucalyptus* plantations in South Africa contribute substantially to the forestry sector, there is limited understanding of the belowground microbial and nematode communities associated with these trees. Plant‐parasitic nematodes are well documented, but the ecological roles of free‐living nematodes, particularly bacterivores that can influence soil microbial dynamics, remain poorly studied. Similarly, while microbiome studies have been undertaken in Brazil and Indonesia, the rhizosphere‐associated microbial diversity of *Eucalyptus* in South Africa has not been systematically investigated. Considering the ecological and economic importance of these plantations, coupled with the increasing need for sustainable forest management, exploring the soil microbiome and nematode interactions is essential for improving productivity, resilience and disease management in South African forestry systems. In addition, *Eucalyptus* is not only economically valuable but also holds cultural and historical significance in South Africa. Many of the earliest plantings, dating back to the 19th century (Rikhotso 2021), have become long‐standing features of the landscape, with certain trees considered iconic or symbolic due to their age and association with settlement history. The endurance of these old *Eucalyptus* stands underscores their role as both ecological and cultural landmarks. Preserving the health and productivity of such plantations, therefore, has value beyond timber production, linking forestry with heritage conservation and sustainable land use. This study aims to characterise the rhizosphere microbiome and nematode communities associated with *Eucalyptus* plantations in South Africa. Specifically, it seeks to (1) document the diversity and composition of microbial and nematode taxa and (2) assess the interactions between nematode abundance, microbial communities and soil physicochemical properties.

## 2 Materials and Methods

### 2.1 Ethics Approval and Consent to Participate

This study did not involve human participants, their data or other personal information and thus, ethics approval and consent to participate are not applicable.

### 2.2 Soil Sampling and Processing

**FIGURE 1:**
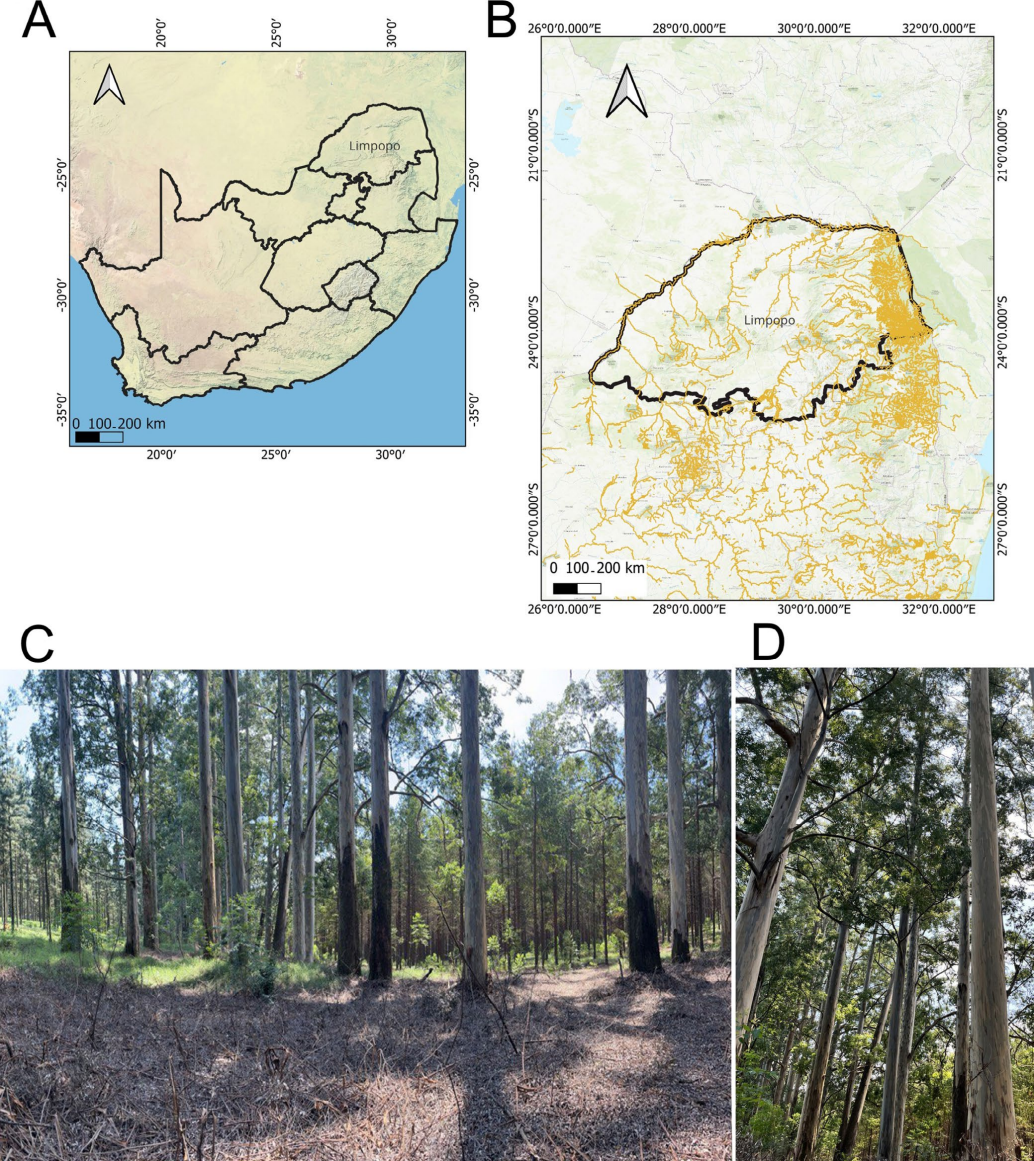
Map of South Africa indicating the location of the *E. saligna* where soil samples were isolated for nematodes and bacteria.

Soil samples were collected from four *Eucalyptus saligna* trees/sites (hereafter EU1–EU4) in Magoebaskloof State Forest, Limpopo Province, South Africa (23°48′50.6″ S, 29°56′58.1″ E) (Figure 1). At each site, we randomly (Shokoohi 2025) collected 10 soil cores (0–35 cm depth) around the target tree; the 10 cores per site were pooled to form one composite sample per site (*n* = 4 composite samples). All 40 individual cores (10 per site) were processed for nematode extraction, while the four site‐level composites were used for 16S rRNA gene profiling. Nematode extraction, fixation and morphological identification followed De Grisse (1969) and standard taxonomic keys (Siddiqi 2000; Andrássy 2005; Castillo and Vovlas 2007; Geraert 2008, 2011; Shokoohi and Abolafia 2019).

### 2.3 Soil Microbiome Analyses

Soil samples obtained from the rhizosphere of *E. saligna* were processed through a series of steps to ensure high‐quality DNA extraction. Total genomic DNA was isolated using the ZymoBIOMICS DNA Miniprep Kit, following the supplier's protocol. The integrity and concentration of the extracted DNA were assessed by agarose gel electrophoresis and a NanoDrop 2000C spectrophotometer. Amplification of the bacterial 16S rRNA gene targeted the V4 hypervariable region, employing the universal primer set 515f (5′‐GTGYCAGCMGCCGCGGTAA‐3′) and 806r (5′‐GGACTACNVGGGTWTCTAAT‐3′), as recommended by Wasimuddin et al. (2020). Each PCR assay was prepared in a 30 μL reaction mixture containing 10–15 ng of template DNA, 12.5 μL of 2× PCR Master Mix Red, 1 μL of each primer (10 pmol/μL) and nuclease‐free water. Amplification was conducted in an Eppendorf Mastercycler Gradient, with the cycling profile consisting of an initial denaturation at 94°C for 3 min; 37 cycles of denaturation at 94°C for 45 s, annealing at 50°C for 45 s and extension at 72°C for 45 s; followed by a final elongation step at 72°C for 6 min and a hold at 4°C. The resulting PCR products were examined on 1.5% agarose gels prepared with TBE buffer (40 mM Tris, 40 mM boric acid, 1 mM EDTA), stained with SafeView (abm, Canada) and visualised under UV illumination. Amplicons were subsequently gel‐purified, end‐repaired and ligated with sequencing adapters. After quantification, each sample was uniquely indexed and further purified using the QIAQuick PCR Purification Kit (Qiagen) in accordance with the manufacturer's recommendations. DNA was eluted in 25 μL of buffer in sterile 1.5 mL microtubes and re‐checked on 2% agarose gels stained with 5 μg/mL SafeView Classic, visualised on a ChemiDoc imaging system. Sequencing was performed on the Illumina MiSeq platform using the MiSeq v3 kit (600 cycles), generating paired‐end reads of 2 × 300 bp, with approximately 20 MB of data produced per sample. All sequencing data have been deposited in the NCBI Sequence Read Archive under BioProject ID PRJNA1321316, with accession numbers SAMN51203710–SAMN51203713.

### 2.4 Microbiome Bioinformatics

Microbiome bioinformatic analyses were carried out using QIIME 2 (version 2017.4) (Bokulich, Dillon, et al. 2018; Bokulich, Kaehler, et al. 2018; Bolyen et al. 2019). Raw sequence reads were processed with the DADA2 pipeline (Callahan et al. 2016) through the q2‐dada2 plugin to remove low‐quality reads and generate high‐resolution amplicon sequence variants (ASVs). Multiple sequence alignment of ASVs was performed using MAFFT (Katoh et al. 2002) via q2‐alignment and phylogenetic trees were inferred with FastTree2 (Price et al. 2010) implemented in q2‐phylogeny. Diversity analyses included alpha‐diversity estimates such as observed feature counts and Faith's phylogenetic diversity index (Faith 1992). These calculations were performed in q2‐diversity following rarefaction of all samples to a uniform depth of 5698 sequences, ensuring comparability across datasets (Lozupone et al. 2007). Taxonomic classification of ASVs was carried out using the q2‐feature‐classifier against the SILVA v132 reference database (McDonald et al. 2012; Bokulich, Dillon, et al. 2018; Bokulich, Kaehler, et al. 2018).

### 2.5 Soil Physicochemical Analysis

Soil chemical characteristics, including ammonia, nitrate and phosphate, were analysed at the Aquaculture Research Unit laboratory. Measurements were conducted primarily with a Hach spectrophotometer (USA), following the manufacturer's guidelines, while soil pH was determined using a Thermo Scientific Orion 3 Star pH Benchtop meter (USA). Soil electrical conductivity (EC) was measured using a YSI conductivity meter (YSI Inc., Yellow Springs, Ohio, USA) in accordance with the manufacturer's instructions. Potassium concentrations were quantified using the APHA (1998) standard procedure, EPA method 200.7. Ammonia was evaluated through methods 1.14752.0001, 1.14752.0002 and 1.00683.0001. Phosphate content was assessed with method 1.14848.0001 and nitrate was measured following the Cadmium reduction technique (method 8171, DOC316.53.01069; Hach 2012). Phosphate and ammonia were additionally confirmed using the USEPA PhosVer 3 protocol (Hach 2012). All chemical analyses were ultimately performed using spectrophotometric techniques to ensure consistent and accurate quantification. Soil texture was analysed using van Capelle et al. (2012).

### 2.6 Relationship of Nematode and Soil Physicochemical

The associations between nematode abundance, bacterial communities and soil physicochemical properties were examined using Pearson's correlation analysis in XLSTAT (Addinsoft 2007). To investigate broader patterns among soil parameters (pH, ammonia, nitrate, phosphate and soil EC) in relation to nematode and bacterial populations, a principal component analysis (PCA) was performed following the methodology of Renčo et al. (2019). Abundances of nematode genera and bacterial taxa were used to ordinate sampling sites within the PCA framework in XLSTAT. Soil factors were included as supplementary variables to assess their contribution to nematode and bacterial distribution. Principal component scores were derived from eigenvalues generated in XLSTAT, with the first two axes (PC1 and PC2) selected to construct a two‐dimensional ordination plot illustrating the main gradients of variation. Pearson's correlation analysis was used to explore linear associations between bacterial taxa, nematode genera and soil physicochemical variables at the site level. This approach assumes approximately linear or monotonic relationships among variables and treats each site‐level composite sample as an independent observational unit. Given the limited number of site‐level replicates, correlation analyses were applied in an exploratory context to identify patterns of co‐variation rather than to infer causality or make definitive statistical inferences. Accordingly, correlation coefficients are interpreted as indicative of potential ecological associations that warrant further validation through studies with increased biological replication or experimental designs.

### 2.7 Statistical Analysis

The relationship between nematode and bacterial population density (MPD) and their frequency of occurrence (FO) was evaluated by calculating the prominence value (PV) for each genus. This approach was used to identify the predominant genera present in the *E. saligna* soils of Magoebaskloof mountain in Limpopo Province. The PV was determined following the formula described by Norton and Schmitt (1978).

$$\mathrm{PV}=\text{Population density}\times \sqrt{\;\text{frequency of occurrence}}$$

Additionally, frequency of occurrence (FO%) = (Number of samples containing a genus/number of total samples) × 100; including those with zero counts for that genus were calculated based on Shokoohi (2023).

Microbiome analyses were based on four site‐level composite samples (EU1–EU4). While this limited the number of independent biological replicates, the composite sampling strategy was employed to capture within‐site spatial heterogeneity by pooling multiple soil cores and to reduce microscale variability. Consequently, correlation, PCA and network analyses were applied in an exploratory and descriptive framework to identify dominant gradients, co‐occurrence patterns and potential hub taxa rather than to test strong statistical hypotheses. The results should therefore be interpreted as indicative patterns at the site level and future studies with increased biological replication will be required to further validate these associations.

The applied statistical approaches were selected to accommodate the limited number of site‐level composite samples while allowing integrated exploration of soil physicochemical variables, bacterial communities and nematode assemblages. Multivariate ordination (PCA), correlation analysis and network analysis are commonly used in exploratory microbiome studies to summarise complex, high‐dimensional data and to identify dominant gradients and co‐occurrence patterns. Given the study design, these methods were employed to provide a descriptive overview of community structure and potential associations rather than to perform formal hypothesis testing.

### 2.8 Data Visualisation

The composition of nematode/bacteria genera across four samples was analysed using Gephi 0.10.1 software (Bastian et al. 2009). Initially, each soil sample location was entered as a site node and spatially arranged for placement. Subsequently, the remaining nodes representing the nematodes identified in this study were added, with their positions determined by the Fruchterman–Reingold layout. This layout arranged nodes based on the strength of their connections to the locked site nodes, resulting in closer proximity for nematode nodes with stronger connections to the site. Additionally, the thickness of the connecting lines and arrows illustrated the strength of these connections. The inherent characteristics of the Fruchterman–Reingold layout also facilitated the identification of singly connected nodes. Network edges were defined based on strong pairwise Pearson correlations among bacterial taxa, nematode genera and soil physicochemical variables, with only associations exceeding a predefined correlation threshold retained. This approach was intended to highlight consistent co‐occurrence patterns while minimising spurious connections arising from small sample sizes. Accordingly, the resulting networks were interpreted as descriptive representations of potential associations rather than evidence of direct interactions.

## 3 Result

### 3.1 Diversity of Bacteria

At the phylum level, Proteobacteria were the most abundant, followed by Acidobacteria, Actinobacteria and Planctomycetes. Other phyla, such as Verrucomicrobia, Chloroflexi, Firmicutes, Bacteroidetes and Gemmatimonadetes, were detected at moderate to low levels (Figure S1). At the order level, the community was dominated by Rhizobiales (Alphaproteobacteria), along with Subgroup 2 (Acidobacteriia), Frankiales (Actinobacteria), Gemmatales (Planctomycetes) and Solibacterales (Acidobacteriia) (Figure S2).

Overall, the bacterial community beneath the *E. saligna* tree was taxonomically diverse, with Proteobacteria (especially Alphaproteobacteria: Rhizobiales and Xanthobacteraceae) forming the ecological backbone, supported by Acidobacteria, Actinobacteria and Planctomycetes. This composition reflects a stable and functionally rich soil microbiome, consistent with taxa known to be involved in nutrient cycling, nitrogen fixation and forest soil functioning.

**FIGURE 2:**
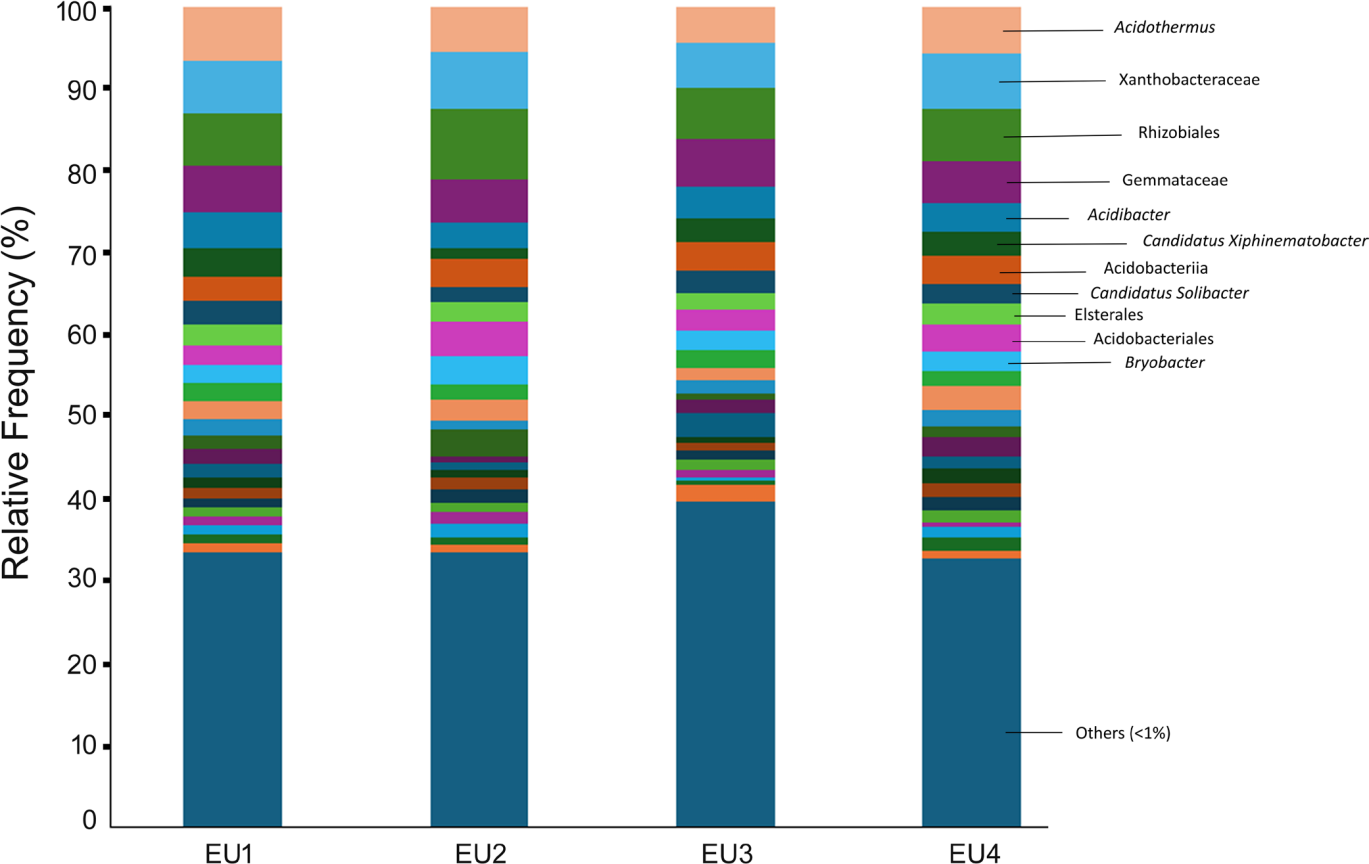
The most abundant bacteria associated with *E. saligna* in Magoebaskloof, Limpopo Province, South Africa.

Across all the sampling points, we identified 11 bacterial genera with the most FO% (= 100) as displayed in Figure 2 (Table 1). Within the subset of taxa detected consistently across all four *E. saligna* sampling sites (FO% = 100%), several genera exhibited high PV, reflecting both their abundance and ecological importance in the rhizosphere.

**TABLE 1:** Characteristics of the dominant bacterial species associated with *Eucalyptus saligna* in Magoebaskloof, Limpopo Province, South Africa.

Genus/uncultured member	Phylum	Order	Gram type
*Bryobacter*	Acidobacteria	Solibacterales	Gram−
Acidobacteriales member	Acidobacteria	Acidobacteriales	Gram−
Elsterales member	Acidobacteria	Elsterales	Gram−
*Candidatus Solibacter*	Acidobacteria	Solibacteriales	Gram−
Acidobacteriia member	Acidobacteria	Subgroup 2	Gram−
*Candidatus Xiphinematobacter*	Verrucomicrobia	Chthoniobacterales	Gram−
*Acidibacter*	Acidobacteria	Gammaproteobacteria	Gram−
Gemmataceae member	Planctomycetota	Gemmatales	Gram−(atypical cell wall)
Rhizobiales member	Proteobacteria	Rhizobiales	Gram−
Xanthobacteraceae member	Proteobacteria	Rhizobiales	Gram−
*Acidothermus*	Actinobacteria	Frankiales	Gram+

**FIGURE 3:**
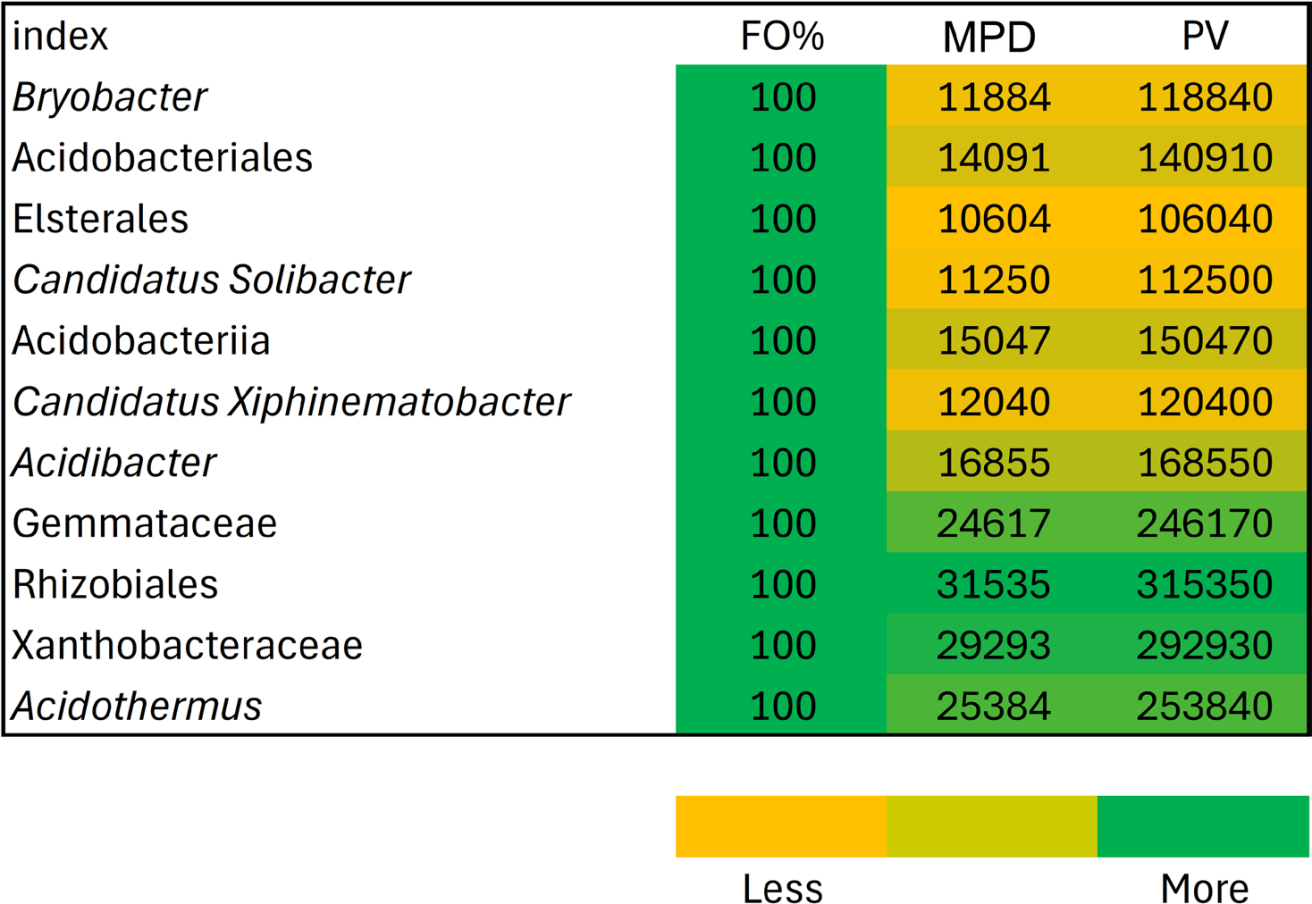
Frequency of Occurrence (FO%) and Prominence Value (PV) of bacterial associated with *E. slaigna* in Magoebaskloof, Limpopo Province, South Africa. [The colour of each column represents the value, ranging from minimum (yellow) to maximum (green)].

Among the Acidobacteria, *Acidibacter* (PV = 168,550), *Bryobacter* (PV = 118,840), *Candidatus Solibacter* (PV = 112,500) and an uncultured Subgroup 2 taxon of Acidobacteria (PV = 150,470) were notable. These taxa indicate the strong representation of Acidobacteria in the *E. saligna* rhizosphere, consistent with their known role in acidic and organic matter–rich soils. Furthermore, *Elsterales* (PV = 106,040) showed high prominence. Among Proteobacteria, members of the Rhizobiales showed particularly high prominence. Notably, the uncultured Rhizobiales taxon recorded the highest PV (315350), followed by Xanthobacteraceae (PV = 292,930). This suggests that Alphaproteobacteria are dominant and potentially functionally significant members of the microbial community (Figure 3).

Other taxa included *Candidatus Xiphinematobacter* (Verrucomicrobia; PV = 120,400), an uncultured Gemmataceae member (Planctomycetes; PV = 246,170) and *Acidothermus* (Actinobacteria; PV = 253,840). These groups, although taxonomically diverse, all demonstrated consistent occurrence and strong ecological contributions within the rhizosphere microbiome (Figure 3).

Together, these results highlight a core microbiome of *E. saligna* rhizosphere soils, dominated by Alphaproteobacteria (Rhizobiales, Xanthobacteraceae) and supported by functionally important Acidobacteria (*Bryobacter*, *Candidatus Solibacter*, Subgroup 2). The presence of taxa such as *Acidibacter*, *Candidatus Xiphinematobacter* and *Acidothermus* further underscores the diverse ecological strategies contributing to microbial adaptation in these soils.

### 3.2 Diversity of Nematodes

Across all the sampling points, we identified 19 nematode genera (Figures S3 and S4). A diverse assemblage of nematodes was recovered from the *E. saligna* rhizosphere, with FO% ranging from 25% to 100% and the prominence value (PV) spanning from 25.0 to 5759.1.

**FIGURE 4:**
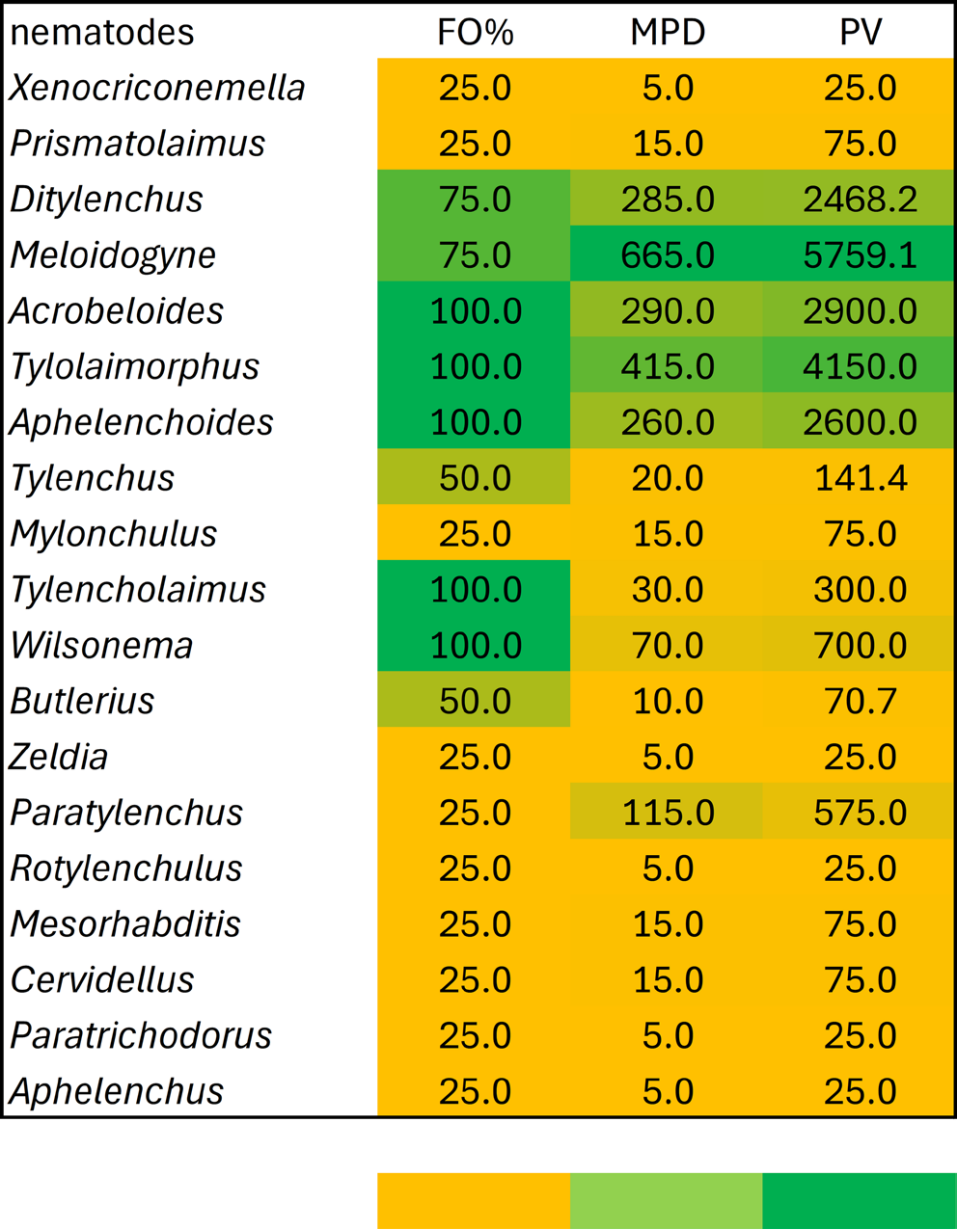
Frequency of Occurrence (FO%) and Prominence Value (PV) of nematodes associated with *E. slaigna* in Magoebaskloof, Limpopo Province, South Africa. [The colour of each column represents the value, ranging from minimum (yellow) to maximum (green)].

Among the taxa with the highest FO% (100%), *Acrobeloides* (PV = 2900.0), *Tylolaimorphus* (PV = 4150.0) and *Aphelenchoides* (PV = 2600.0) emerged as dominant genera. Other consistently present taxa included *Tylencholaimus* (PV = 300.0) and *Wilsonema* (PV = 700.0), which, although less prominent, still represented widespread components of the nematode community (Figure 4).

Within the group of nematodes occurring at 75% FO, *Ditylenchus* (PV = 2468.2) and *Meloidogyne* (PV = 5759.1) were particularly noteworthy. *Meloidogyne* recorded the highest PV overall, indicating that plant‐parasitic nematodes (PPNs) play a major role in shaping soil nematode assemblages associated with *E. saligna* .

Nematodes with intermediate FO% (50%), such as *Tylenchus* (PV = 141.4) and *Butlerius* (PV = 70.7), were moderately represented across sites. In contrast, several genera (*Xenocriconemella, Prismatolaimus, Mylonchulus, Zeldia, Paratylenchus, Rotylenchulus, Mesorhabditis, Cervidellus, Paratrichodorus, Aphelenchus*) were detected only sporadically (FO% = 25%) and exhibited low PV values (25.0–575.0), indicating they are rare or site‐specific taxa (Figure 4).

Overall, the nematode community of the *E. saligna* rhizosphere was characterised by a dominant core group comprising both plant‐parasitic nematodes (*Meloidogyne*, *Ditylenchus*, *Paratylenchus*, *Rotylenchulus*) and free‐living taxa (*Acrobeloides*, *Tylolaimorphus*, *Aphelenchoides*), alongside a diverse assemblage of less frequent genera that contribute to soil ecological heterogeneity (Figures S1 and S2; Table 2).

**TABLE 2:** Characteristics of the nematode species associated with *Eucalyptus saligna* in Magoebaskloof, Limpopo Province, South Africa.

Nematode	C–p class	P–p class	Feeding type	Mass, μg
*Xenocriconemella*	0	3	Herbivores—ectoparasites	0.567
*Meloidogyne*	0	3	Herbivores—sedentary parasites	92.064
*Paratrichodorus*	0	4	Herbivores—ectoparasites	0.747
*Paratylenchus*	0	2	Herbivores—ectoparasites	0.051
*Rotylenchulus*	0	3	Herbivores—sedentary parasites	1.77
*Tylenchus*	2	0	Fungivores	0.36
*Aphelenchoides*	2	0	Fungivores	0.151
*Aphelenchus*	2	0	Fungivores	0.218
*Ditylenchus*	2	0	Fungivores	0.451
*Tylolaimorphus*	4	0	Fungivores	42.765
*Acrobeloides*	2	0	Bacterivores	1.263
*Cervidellus*	2	0	Bacterivores	0.174
*Mesorhabditis*	1	0	Bacterivores	0.568
*Prismatolaimus*	3	0	Bacterivores	0.357
*Wilsonema*	2	0	Bacterivores	0.061
*Zeldia*	2	0	Bacterivores	0.717
*Mylonchulus*	4	0	Predators	11.588
*Butlerius*	1	0	Predators	1.3
*Tylencholaimus*	5	0	Omnivores	6.546

### 3.3 Soil Relationship With Organism Abundance: Bacteria

**FIGURE 5:**
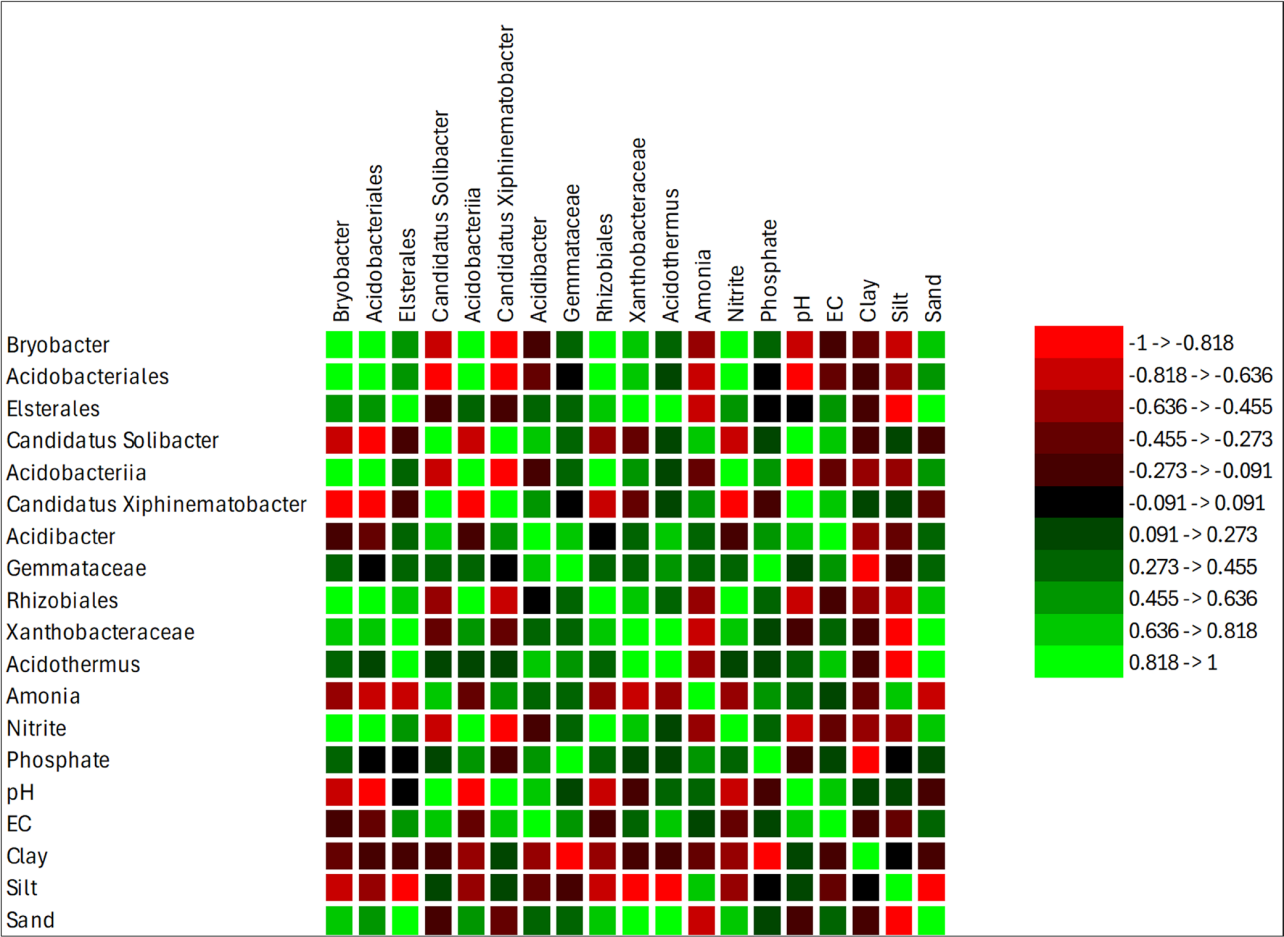
A Pearson correlation showing the relationship between the most abundant bacteria and soil physicochemical properties in *E. saligna* in Magoebaskloof, Limpopo Province, South Africa.

Pearson correlation analysis revealed strong and consistent associations between bacterial groups and soil physicochemical variables (Figure 5). Several taxa, including *Bryobacter*, *Acidobacteriales*, *Acidobacteriia* and Rhizobiales, were highly inter‐correlated (*r* = 0.89–0.99), indicating that these lineages frequently co‐occur in the *Eucalyptus* rhizosphere. Soil texture emerged as a major reason for bacterial abundance. Sand content showed very strong positive correlations with *Elsterales* (*r* = 0.98), *Xanthobacteraceae* (*r* = 0.99) and *Acidothermus* (*r* = 0.89), whereas these taxa were negatively correlated with silt (*r* = −0.88 to −0.99). This confirms the PCA results, suggesting that sandy soils favour Alphaproteobacteria and Actinobacteria, while silty soils constrain their abundance.

Soil pH and nitrogen forms also displayed distinct relationships with bacterial groups. *Candidatus Solibacter* was strongly and positively associated with soil pH (*r* = 0.94) and ammonia (*r* = 0.65), highlighting its adaptation to less acidic, nitrogen‐rich conditions. Similarly, *Candidatus Xiphinematobacter* correlated positively with pH (*r* = 0.99) but negatively with nitrite (*r* = −0.88), suggesting that alkaline soils may favour its persistence. By contrast, *Bryobacter*, Rhizobiales and *Acidobacteriia* correlated strongly with nitrite concentrations (*r* = 0.98–0.99), indicating that these taxa respond to different nitrogen forms than *Candidatus Solibacter*.

Soil EC was positively associated with *Acidibacter* (*r* = 0.95) and *Acidothermus* (*r* = 0.77), suggesting that salinity gradients also contribute to shaping community composition. Collectively, these correlations indicate that soil texture, pH and nitrogen availability are strongly associated with variation in bacterial assemblages in *E. saligna* soils, with different taxa responding to distinct nutrient and edaphic gradients.

**FIGURE 6:**
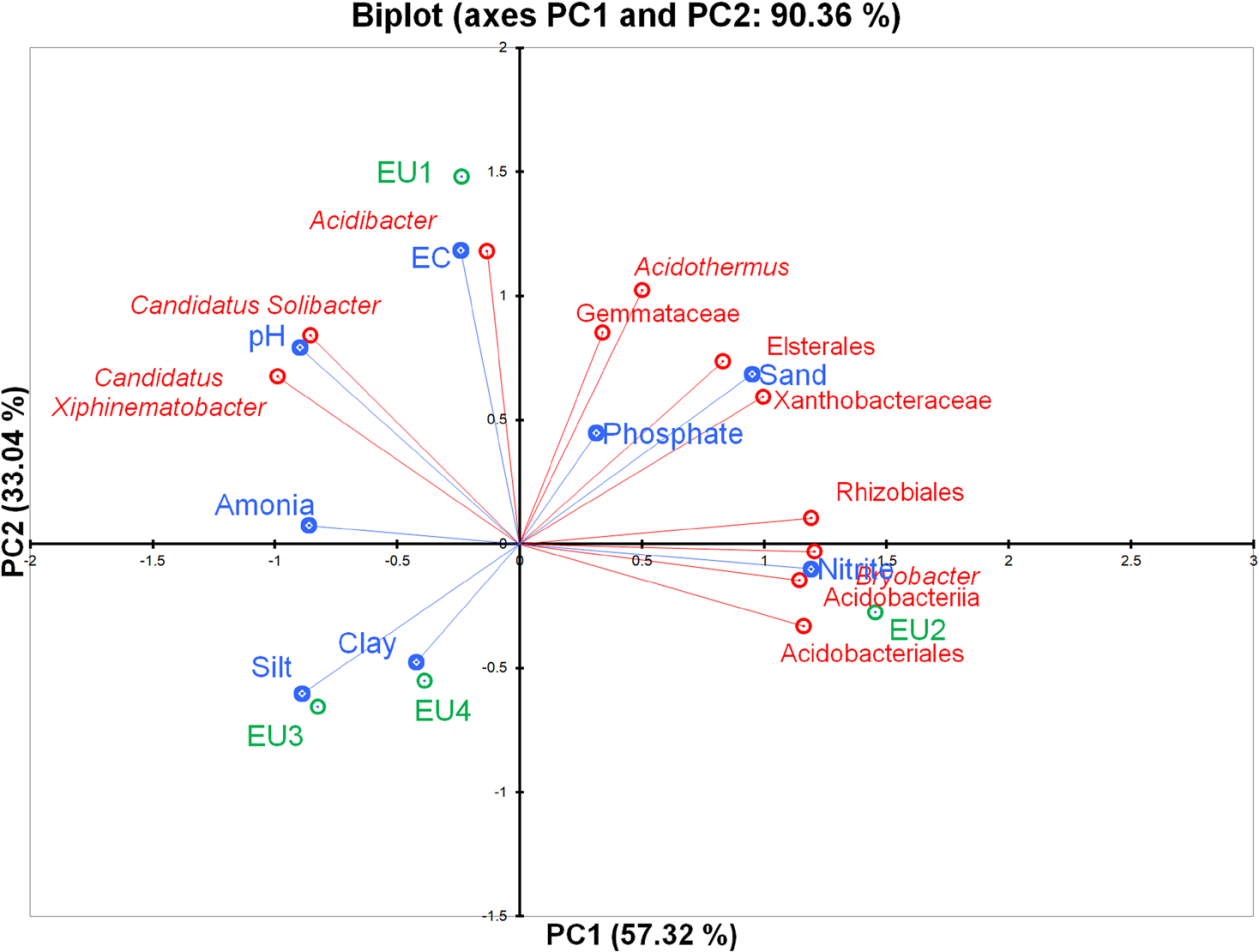
Principal component analysis (PCA) plot showing the relationship between the most abundant bacteria and soil physicochemical properties in *E. saligna* in Magoebaskloof, Limpopo Province, South Africa.

The PCA revealed strong relationships between bacterial groups and soil properties in *E. saligna* soils (Figure 6). The first two principal components (PC1 and PC2) explained 90.36% of the total variance, with PC1 accounting for 57.32% and PC2 for 33.04%.

Along PC1, bacterial groups such as *Bryobacter*, Rhizobiales, Acidobacteriales and Acidobacteriia were positively associated with sand content, nitrite and phosphate, indicating a preference for sandy, nutrient‐rich soils. In contrast, *Candidatus Solibacter* and *Candidatus Xiphinematobacter* were positioned negatively along this axis, showing stronger associations with silt, pH and ammonia, which reflects bacterial adaptation to more silty, less acidic soils with higher ammonia availability. Along PC2, soil EC aligned positively with *Acidibacter*, *Acidothermus* and Gemmataceae, highlighting the importance of salinity in shaping community structure.

Overall, the biplot demonstrates that soil texture (sand, silt, clay), nutrient availability (nitrite, phosphate, ammonia), salinity (EC) and pH were strongly associated with patterns of bacterial community structure in *E. saligna* soils. Bacterial groups clustered according to their ecological preferences, reflecting niche partitioning in response to soil heterogeneity.

### 3.4 Soil Relationship With Organism Abundance: Nematode

**FIGURE 7:**
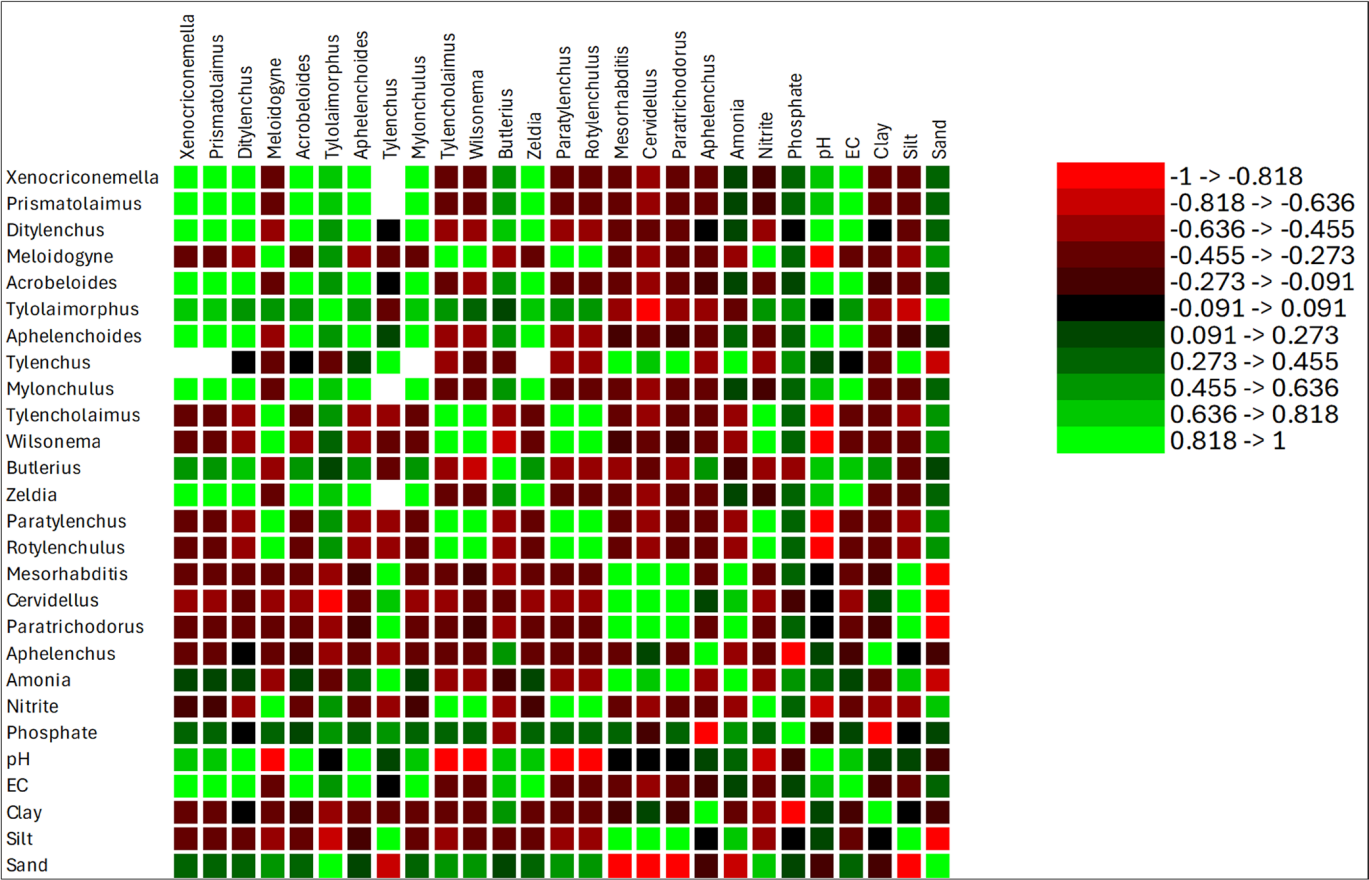
A Pearson correlation showing the relationship between the nematodes and soil physicochemical properties in *E. saligna* in Magoebaskloof, Limpopo Province, South Africa.

Pearson correlation analysis showed distinct associations between nematode taxa and soil physicochemical variables (Figure 7). Several bacterivorous taxa, including *Xenocriconemella*, *Prismatolaimus*, *Mylonchulus*, *Zeldia* and *Acrobeloides*, were highly correlated with each other (*r* = 0.95–1.00), indicating frequent co‐occurrence in the *Eucalyptus* rhizosphere. In contrast, plant‐parasitic nematodes such as *Meloidogyne*, *Tylencholaimus*, *Rotylenchulus* and *Paratylenchus* were negatively correlated with these bacterivores (*r* = −0.33 to −0.61), suggesting contrasting habitat preferences.

Soil texture exerted a strong influence on nematode community structure. Sand content correlated positively with *Tylolaimorphus* (*r* = 0.89), *Tylencholaimus* (*r* = 0.61) and *Meloidogyne* (*r* = 0.58), while silt content showed the opposite trend, being positively related to *Mesorhabditis* (*r* = 0.95) and *Cervidellus* (*r* = 0.97) but negatively related to *Tylolaimorphus* and *Meloidogyne* (*r* = −0.79 to −0.50). This highlights a soil texture gradient where sandy soils were more frequently associated with certain plant‐parasitic and omnivorous taxa, whereas silty soils supported enrichment opportunists such as *Mesorhabditis*.

Soil chemical variables also shaped nematode assemblages. pH correlated positively with *Aphelenchoides* (*r* = 0.83), *Ditylenchus* (*r* = 0.89) and *Xenocriconemella* (*r* = 0.76), while showing a strong negative correlation with *Meloidogyne* (*r* = −0.87). Soil EC was strongly associated with *Acrobeloides* (*r* = 0.99), *Aphelenchoides* (*r* = 0.95) and *Zeldia* (*r* = 0.99), indicating that bacterial‐feeding nematodes thrive in soils with higher ionic content. Nitrogen forms exhibited contrasting patterns: ammonia correlated positively with *Tylenchus* (*r* = 0.98) and *Mesorhabditis* (*r* = 0.86), while nitrite was strongly associated with *Meloidogyne* (*r* = 0.99), *Tylencholaimus* (*r* = 0.99) and *Wilsonema* (*r* = 0.98).

Overall, these patterns indicate that nematode genera show strong associations with soil texture, pH and nitrogen availability. Sandy soils and nitrite enrichment favoured plant‐parasitic taxa such as *Meloidogyne* and *Rotylenchulus*, while silty soils and ammonia enrichment supported enrichment opportunists and bacterivores such as *Mesorhabditis*.

**FIGURE 8:**
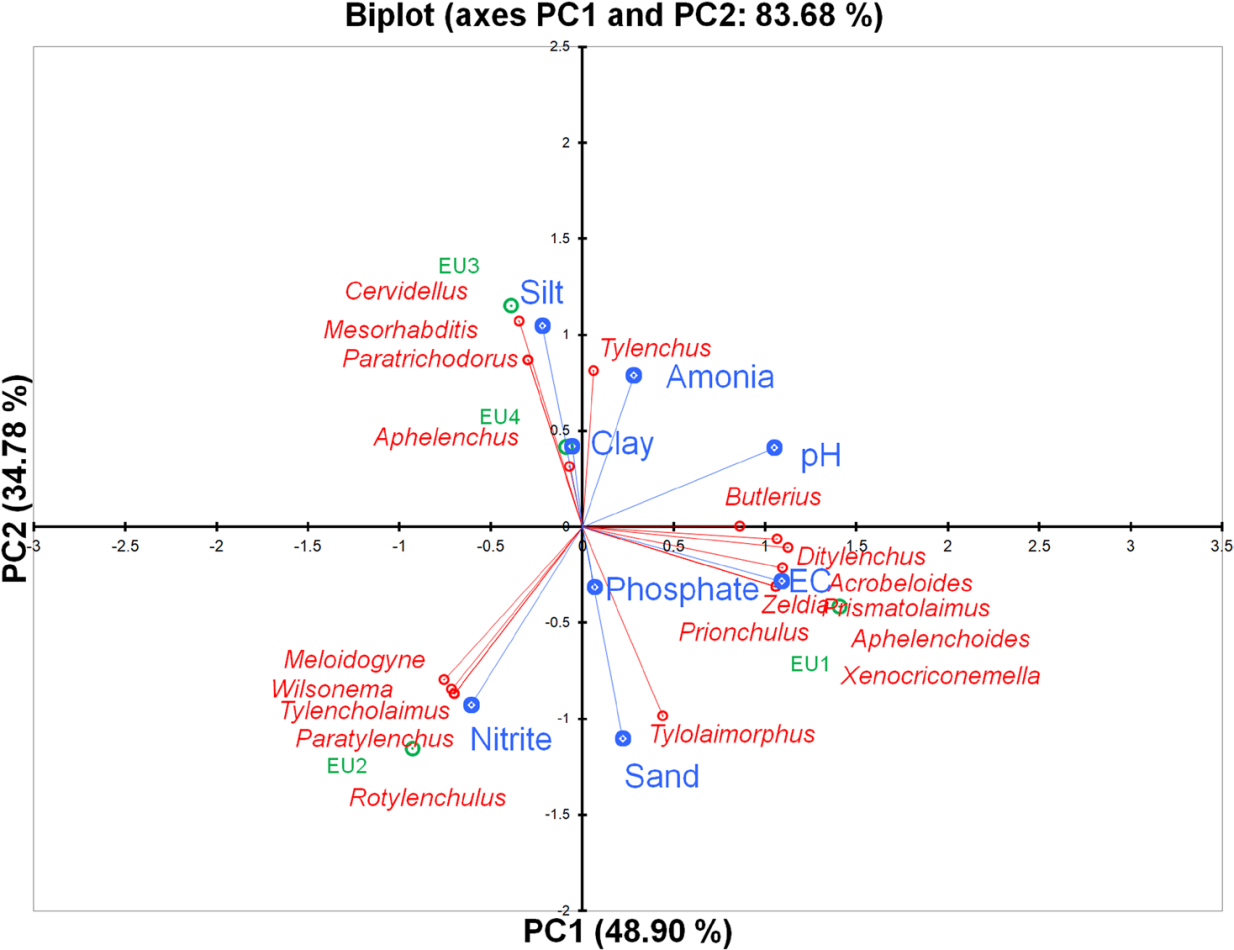
Principal component analysis (PCA) plot showing the relationship between nematodes and soil physicochemical properties in *E. saligna* in Magoebaskloof, Limpopo Province, South Africa.

The PCA explained 83.68% of the total variance, with PC1 accounting for 48.90% and PC2 for 34.78% (Figure 8).

Along PC1, nematode taxa such as *Aphelenchoides*, *Ditylenchus*, and *Xenocriconemella* clustered positively together with soil pH, phosphate and sand content, indicating their preference for sandy, nutrient‐rich and moderately alkaline soils. *Butlerius* was also positioned close to these variables, reflecting a similar ecological association. Conversely, *Meloidogyne*, *Rotylenchulus*, and *Paratylenchus* showed strong negative correlations with PC1, aligning with nitrite, suggesting an adaptation to more nitrite‐enriched soil conditions.

Along PC2, *Aphelenchus*, and *Cervidellus* grouped positively with silt and ammonium, reflecting their affinity for fine‐textured, ammonium‐rich soils. In contrast, *Tylolaimorphus* was negatively associated with this axis, aligning more with sandy soils and nitrite availability.

Overall, the PCA indicates that soil texture (sand, silt, clay), nutrient availability (phosphate, ammonium, nitrite) and pH were strongly associated with patterns of nematode community structure in *E. saligna* soils. Distinct functional groups of nematodes showed clear ecological preferences, with plant‐parasitic taxa generally associated with sandy, phosphate‐rich soils, while other groups were linked to ammonium or nitrite‐enriched environments.

### 3.5 Comparative Results of PCA: Bacteria vs. Nematodes

The PCA analyses revealed that both bacterial and nematode communities in *E. saligna* soils are strongly structured by soil physicochemical properties, although the two groups respond differently to specific associations.

For bacteria, the main structuring factors were soil texture (sand, silt, clay), pH, salinity (EC) and nutrients (nitrite, phosphate, ammonia). Bacterial groups such as *Bryobacter*, Rhizobiales and *Acidobacteriales* were associated with sandy, nitrite‐rich soils, whereas *Candidatus Solibacter* and *Candidatus Xiphinematobacter* were more abundant in silty soils with higher pH and ammonia. High EC favoured *Acidibacter* and *Acidothermus*, indicating that salinity also played a key role in bacterial distribution.

For nematodes, soil texture and nutrient status were also decisive. Plant‐parasitic nematodes such as *Xenocriconemella* was strongly associated with sand, phosphate and higher pH, reflecting a preference for well‐drained, nutrient‐enriched conditions. In contrast, Meloidogyne, and *Rotylenchulus* were linked to nitrite availability, while fungal‐feeding taxa (*Aphelenchus*, *Cervidellus*) were positively associated with silt and ammonium.

Taken together, the results show that soil texture and nutrient gradients are the dominant forces structuring both bacterial and nematode communities, but with contrasting ecological patterns. While bacterial groups appear more sensitive to salinity and nitrogen forms (nitrite, ammonia), nematode assemblages are more closely linked to phosphate and soil texture. These findings suggest that bacteria and nematodes occupy complementary niches in *E. saligna* soils, with bacteria responding rapidly to chemical properties, such as EC and nematodes reflecting broader soil fertility and structural conditions, such as phosphorus and sand.

### 3.6 Network Analysis

The network analysis of nematode–bacteria co‐occurrence across *E. saligna* soils revealed distinct patterns of connectivity among the four sampling sites (EU1–EU4). All sites acted as central hubs in the network, with EU1 showing the highest connectivity (degree = 23; weighted degree = 56,108) and the strongest eigenvector centrality (1.0), indicating its dominant role in structuring community interactions. EU2–EU4 also exhibited high degrees (19–21) and strong eigenvector values (0.90–0.97), confirming that the sites were all well connected to the broader interaction network. Notably, the clustering coefficient of all sites was zero, suggesting that sites primarily functioned as bridging nodes rather than forming tightly knit clusters.

**FIGURE 9:**
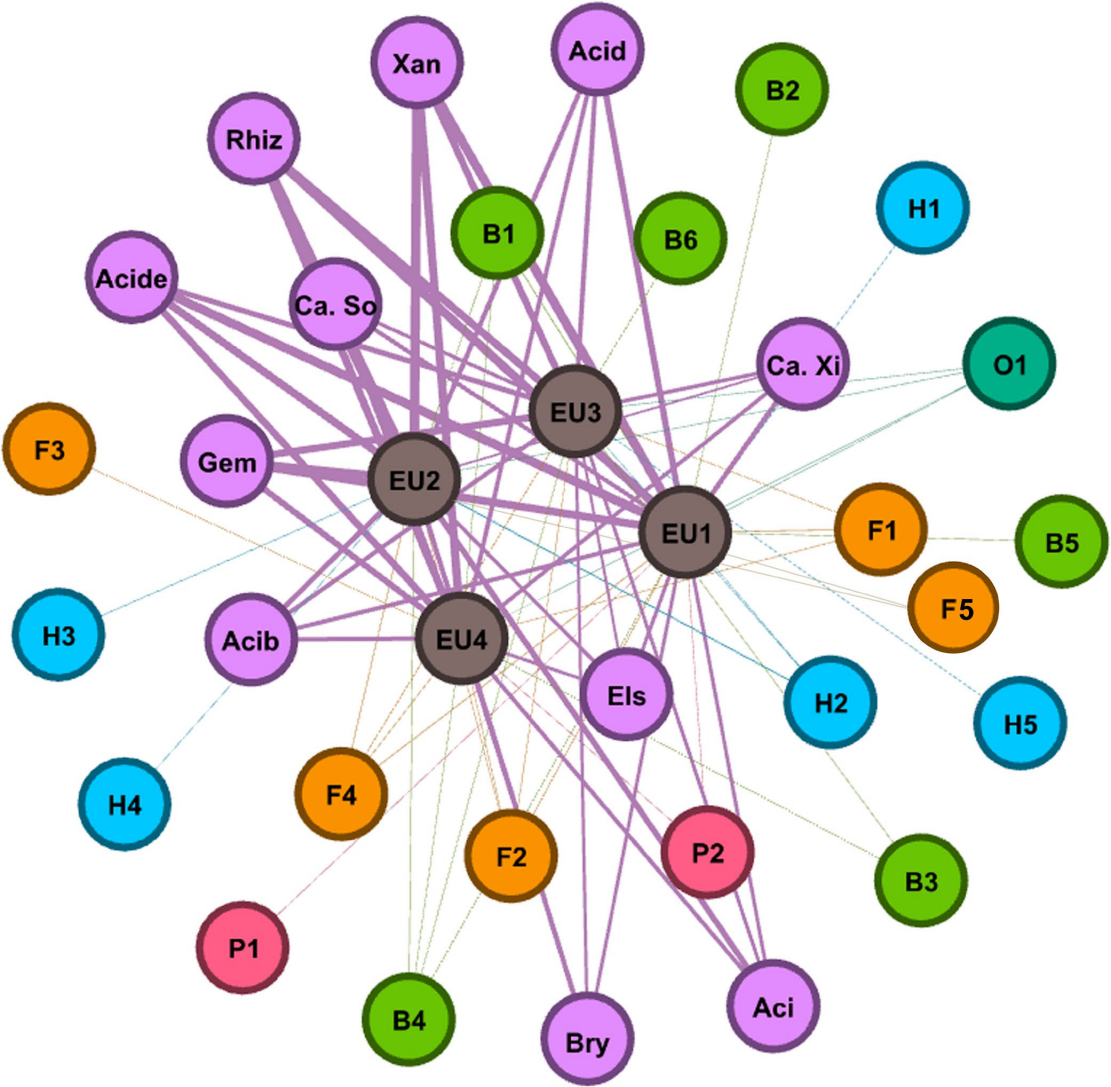
Network analysis showing co‐occurrence relationship between the most abundant bacteria and nematodes in *E. saligna* in Magoebaskloof, Limpopo Province, South Africa. Bry, *Bryobacter*; Aci, Acidobacteriales; Els, Elsterales; Ca. So, *Candidatus Solibacter*; Acib, Acidobacteriia; Ca. Xi, *Candidatus Xiphinematobacter*; Acid, *Acidibacter*; Gem, Gemmataceae; Rhiz, Rhizobiales; Xan, Xanthobacteraceae; Acide, Acidothermus; H1, *Xenocriconemella*; H2, *Meloidogyne*; H3, *Paratylenchus*; H4, *Rotylenchulus*; H5, *Paratrichodorus*; B1, *Acrobeloides*; B2, *Zeldia*; B3, *Cervidellus*; B4, *Wilsonema*; B5, *Prismatolaimus*; B6, *Mesorhabditis*; F1, *Ditylenchus*; F2, *Aphelenchoides*; F3, *Aphelenchus*; F4, *Tylenchus*; F5, *Tylolaimorphus*; O1, *Tylencholaimus*; P1, *Mylonchulus*; P2, *Butlerius*.

Among nematodes, several taxa emerged as key mediators of co‐occurrence with bacteria. *Acrobeloides* (B1), *Wilsonema* (B4), *Aphelenchoides* (F2), *Tylenchus* (F4) and *Tylencholaimus* (O1) demonstrated the highest connectivity (degree = 4 each) and moderate eigenvector centrality (~0.45), highlighting their importance in linking bacteria with sampling sites. Other nematodes, such as *Ditylenchus* (F1) and *Meloidogyne* (H2), showed intermediate degrees (3), whereas *Cervidellus* (B3), *Butlerius* (P2) and *Rotylenchulus* (H4) had low degrees (1–2) and low centrality, reflecting more marginal roles in the network. Similar to the sites, nematodes exhibited zero clustering, indicating that their interactions were mainly direct with bacteria and sites rather than with one another (Figure 9).

Bacterial taxa showed consistently high levels of connectivity within the co‐occurrence network. All examined genera, including *Bryobacter*, Acidobacteriales, Elsterales, *Candidatus Solibacter*, Acidobacteria, *Candidatus Xiphinematobacter*, *Acidibacter*, Gemmataceae, Rhizobiales, Xanthobacteraceae and *Acidothermus*, displayed identical degrees (degree = 4) and moderate eigenvector centrality (0.45), indicating their equivalent roles as central co‐occurrence partners across *E. saligna* sites. However, their weighted degrees varied substantially, with Rhizobiales (31535), Xanthobacteraceae (29293), *Acidothermus* (25384) and Gemmataceae (24617) exhibiting the highest values. This suggests that these genera represent the most strongly interacting bacterial hubs, forming key associations with nematodes and sites. In contrast, genera such as Elsterales (10604), *Candidatus Solibacter* (11250) and *Bryobacter* (11884) showed lower weighted degrees, reflecting weaker but still consistent involvement in the interaction network.

Importantly, all bacterial nodes had a clustering coefficient of zero, confirming that interactions were spread across multiple nodes rather than forming tightly bound bacterial subgroups. This indicates that bacterial co‐occurrence patterns are diffuse and integrative, with multiple taxa bridging nematode–site interactions rather than clustering into isolated bacterial communities.

Overall, the network structure suggests that nematode–bacteria associations in *E. saligna* soils are site‐association and diffuse, with highly connected nematode taxa serving as key bridging nodes. The absence of clustering further emphasises the role of *E. saligna* sites as central connectors in shaping microbial–faunal co‐occurrence patterns, rather than supporting localised, tightly clustered communities.

## 4 Discussion

This study provides one of the first integrated views of bacterial and nematode assemblages in the rhizosphere of *E. saligna* in South Africa, revealing how soil properties and key taxa shape belowground biodiversity. The dominance of Proteobacteria, Acidobacteriota and Actinobacteriota aligns with patterns commonly reported for forest and plantation soils (Fonseca et al. 2018; Lelana et al. 2022; Huo et al. 2024). Although the limited number of site‐level replicates restricts statistical power, the consistency of dominant taxa across all sites and the high variance explained by the first PCA axes suggest that the observed patterns reflect robust ecological signals at the site scale. Accordingly, all reported relationships are based on correlations and multivariate associations and should therefore be interpreted as indicative of co‐variation rather than evidence of causality. It should be mentioned that functional interpretations are inferred from taxonomic identity, ecological guild classification and published literature, as functional processes were not directly measured in this study.

In particular, Rhizobiales and Xanthobacteraceae emerged as central bacterial groups, reflecting their well‐documented roles in nitrogen cycling and plant–microbe interactions. Similarly, Acidobacteria genera such as *Bryobacter* and *Candidatus Solibacter* were abundant, highlighting their adaptation to acidic, organic‐rich soils typical of forest environments.

The genus *Acidothermus* comprises thermophilic, acidophilic bacteria with the ability to degrade cellulose, thereby contributing to nutrient turnover and soil enrichment (Wang et al. 2015). Previous research has reported the occurrence of this genus in association with *Eucalyptus pellita* in Indonesia. Given that the *Eucalyptus* plantations in Limpopo Province, South Africa, are established adjacent to pine forests—and in some areas even replacing them—the presence of *Acidothermus* is consistent with soil conditions known to support acidophilic and cellulolytic taxa, as reported for forest‐influenced systems (Fan et al. 2023).

Among the bacterial taxa identified, members of the genus *Bryobacter* are recognised for their role in lignin and cellulose degradation (Mikhail et al. 2021). In this study, *Bryobacter* was detected for the first time in association with *Eucalyptus*, whereas previous reports linked it to tea plantations in China (Zhao et al. 2024). In the Magoebaskloof region, the accumulation of *Eucalyptus* leaf litter likely provides substrates compatible with its reported lignin and cellulose degradation capacity, suggesting a potential indirect influence on soil microbial habitat conditions.

The genus *Candidatus Solibacter* was also detected in the present study, consistent with earlier findings from Indonesia, where it was associated with *E. pellita* (Lelana et al. 2022). This genus has been widely associated with cellulose decomposition and nitrogen cycling in forest soils (Ward et al. 2009).

The detection of *Candidatus Xiphinematobacter* in the absence of its typical host, *Xiphinema* nematodes, underscores a notable association between plant‐parasitic nematodes and *Eucalyptus* ecosystems. This bacterium is recognised as an endosymbiont of *Xiphinema* species (Shokoohi and Masoko 2024). To our knowledge, this is the first report of *Candidatus Xiphinematobacter* within *Eucalyptus* plantations in South Africa. Its detection may indicate soil conditions compatible with *Xiphinema* occurrence, warranting further investigation, which is well known for its detrimental effects on root integrity and overall plantation performance. These findings highlight the need for further research into soil microbial communities and their interactions with nematodes, with particular emphasis on mitigating potential risks to *Eucalyptus* health and productivity.

The prominence of *Meloidogyne* highlights the potential importance of plant–parasitic nematodes as threats to *Eucalyptus* productivity, aligning with previous reports of root‐knot nematodes as key pests in *Eucalyptus* systems (Lopes Vieira et al. 2024). *Meloidogyne* species were previously reported in association with *Eucalyptus* in Limpopo Province (Marais and Swart 2003). Conversely, bacterivorous and fungivorous nematodes detected in this study are widely reported to participate in microbial grazing and nutrient mineralisation, suggesting potential roles in soil food‐web dynamics (Haraguchi and Yoshiga 2020; Shokoohi et al. 2022, 2024; Shokoohi 2023).

Soil physicochemical properties emerged as strong filters of both bacterial and nematode distributions. Bacterial and nematode communities exhibited contrasting associations with soil physicochemical gradients. This divergence suggests complementary ecological strategies, where microbes respond rapidly to chemical gradients and nematodes reflect broader fertility and structural conditions. Such patterns are in line with earlier studies emphasising the role of edaphic factors in driving soil biota distributions (Renčo et al. 2019). The prior research conducted by Huo et al. (2024) demonstrated a lack of correlation between soil pH levels and the abundance of *Candidatus solibacter*, suggesting a consistent pattern across the study sites. However, the results from the current investigation reveal a significant relationship between these two variables, indicating a complex interaction that warrants further exploration. This apparent contradiction may stem from several contextual factors, particularly the geographical location where the *Eucalyptus* species were studied, as well as the inherent differences in soil types found in these areas. Variability in environmental conditions, such as climate and land use practices, could also influence microbial communities, further contributing to the observed discrepancies. Thus, understanding the specific local conditions and soil characteristics is essential for interpreting the interactions between pH and *Candidatus solibacter* in these diverse ecosystems. Previous research on bacterial communities in *Eucalyptus* soils in Brazil (Fonseca et al. 2018) indicated that soil texture, particularly the proportion of sand, positively influences bacterial diversity. Consistent with these findings, the present study observed a similar effect.

Network analysis suggests that *Eucalyptus* sites function as hubs, integrating nematode–bacteria co‐occurrence rather than supporting tightly bound clusters. The absence of clustering coefficients suggests diffuse and cross‐site associations, with connector taxa such as Rhizobiales, *Xanthobacteraceae*, *Acidothermus* and nematodes like *Acrobeloides* and *Wilsonema* occupying central positions within co‐occurrence networks, which may indicate potential roles in ecosystem connectivity based on network theory. These findings suggest that soil biodiversity in *Eucalyptus* plantations is characterised by flexible, cross‐linking co‐occurrence patterns rather than isolated guilds that may enhance resilience against environmental fluctuations.

## 5 Study Limitations

This study was based on four site‐level composite soil samples (EU1–EU4), which constrained the number of independent biological replicates available for microbiome analyses. Although composite sampling is a common and practical approach in soil ecology to capture within‐site heterogeneity and reduce microscale variability, the limited replication reduces the statistical power of inferential analyses. Consequently, correlation analyses, PCA and network analyses were applied in an exploratory framework to identify dominant gradients, co‐occurrence patterns and potential hub taxa rather than to establish causal relationships.

In addition, the cross‐sectional nature of the sampling provides a snapshot of bacterial and nematode communities at a single time point. Therefore, observed associations between soil physicochemical properties, microbial taxa and nematode assemblages should be interpreted as indicative patterns rather than evidence of direct causation. Future studies incorporating increased numbers of independent sites, temporal replication and experimental manipulations will be necessary to validate the inferred interactions and to test causal mechanisms underlying nematode–microbe–soil relationships in *Eucalyptus* rhizospheres.

The limited number of independent site‐level replicates may also contribute to an overestimation of effect sizes in correlation coefficients, ordination loadings and inferred network connections, a phenomenon commonly reported in small‐sample ecological studies. Consequently, the magnitude of observed associations should be interpreted cautiously, with emphasis placed on consistent patterns across analyses rather than on absolute effect sizes. Validation of these associations through increased replication, temporal sampling or experimental manipulation will be necessary to confirm their ecological relevance.

## 6 Conclusion

This study offers the first integrated view of bacterial and nematode assemblages in *E. saligna* rhizospheres in South Africa, revealing how soil properties and key taxa represent belowground biodiversity. Dominant bacterial groups (Proteobacteria, Acidobacteria, Actinobacteria, Planctomycetes) and central taxa such as Rhizobiales, Xanthobacteraceae, *Acidothermus*, *Bryobacter* and *Candidatus Solibacter* are commonly associated in the literature with nutrient cycling and organic matter decomposition, suggesting potential functional relevance in *Eucalyptus* soils. The unexpected detection of *Candidatus Xiphinematobacter* highlights novel plant–nematode–microbe interactions. Nematode communities, including both plant‐parasitic (*Meloidogyne*, *Paratylenchus*) and free‐living taxa (*Acrobeloides*, *Aphelenchoides*), illustrate their dual role in threatening productivity and sustaining soil health. Soil pH, texture and nutrient content emerged as key variables associated with, while network analysis revealed diffuse, cross‐site co‐occurrence patterns with Connector taxa occupying central positions within co‐occurrence networks, potentially contributing to ecosystem connectivity. These findings highlight distinct co‐occurrence patterns between microbial and nematode communities, which—based on known functional roles reported in the literature—may have implications for soil functioning. This integrated perspective provides a critical foundation for advancing sustainable plantation practices and soil biodiversity conservation. Therefore, future research should focus on elucidating the functional roles and interactions of key bacterial and nematode taxa in *Eucalyptus* rhizospheres to inform sustainable soil management and enhance plantation resilience.

## Author Contributions

E.S. and P.M. conceptualised the study. E.S. designed and carried out experiments, analysed data and wrote the manuscript. E.S. and P.M. revised the manuscript. All authors approved the final version of the manuscript.

## Funding

This work was supported by the University of Limpopo (RNA‐2022).

## Ethics Statement

This study did not involve human participants, their data, or other personal information and thus, ethics approval and consent to participate are not applicable.

## Consent

The authors have nothing to report.

## Conflicts of Interest

The authors declare no conflicts of interest.

## Supporting information


**Figure S1:** Dominant bacterial phyla associated with *Eucalyptus saligna* .
**Figure S2:** Dominant bacterial orders associated with *Eucalyptus saligna* .
**Figure S3:** Representative genera of mostly Plant‐parasitic nematodes.
**Figure S4:** Representative genera of mostly Free‐living nematodes.

## Data Availability

All relevant data are within the manuscript and its Supporting Information files. The data for the microbiome were deposited and available in the NCBI GenBank. The microbiome of *Eucalyptus* soils was deposited in the Biosamples under the accession numbers: SAMN51203710–SAMN51203713.
