# Supplementary material for: Soil Biodiversity of Eucalyptus saligna: Insights Into Bacterial and Nematode Communities
Source: Environ Microbiol Rep. 2026 Apr 8;18(2):e70341. doi: 10.1111/1758-2229.70341 (PMC13061582; doi:10.1111/1758-2229.70341)
Supplement: Supplementary file 1 — Figure S1: Dominant bacterial phyla associated with Eucalyptus saligna . Figure S2: Dominant bacterial orders associated with Eucalyptus saligna . Figure S3: Representative genera of mostly Plant‐parasitic nematodes. Figure S4: Representative genera of mostly Free‐living nematodes. [file EMI4-18-e70341-s001.docx]

**Soil Biodiversity of *Eucalyptus saligna*: Insights into Bacterial and Nematode Communities**

**Ebrahim Shokoohi^1^,** **and Peter Masoko^1^**

*^1^Department of Biochemistry, Microbiology, and Biotechnology, University of Limpopo, Private Bag X1106, Sovenga, 0727, South Africa.*

Corresponding author e-mail: Ebrahim.shokoohi@ul.ac.za

Supplementary


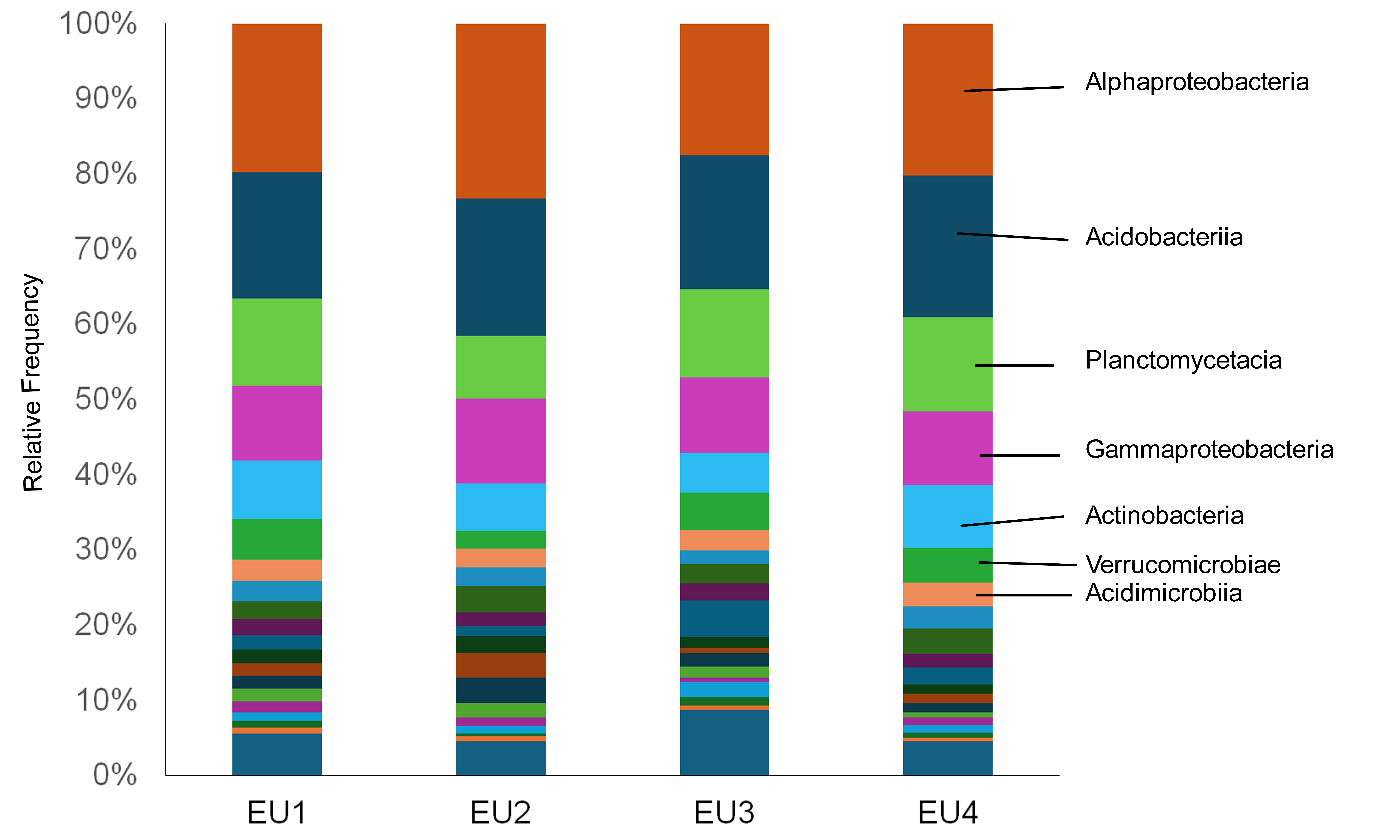


**FIGURE S1**. The most abundant bacterial phyla associated with *E. saligna* in Magoebaskloof, Limpopo Province, South Africa.


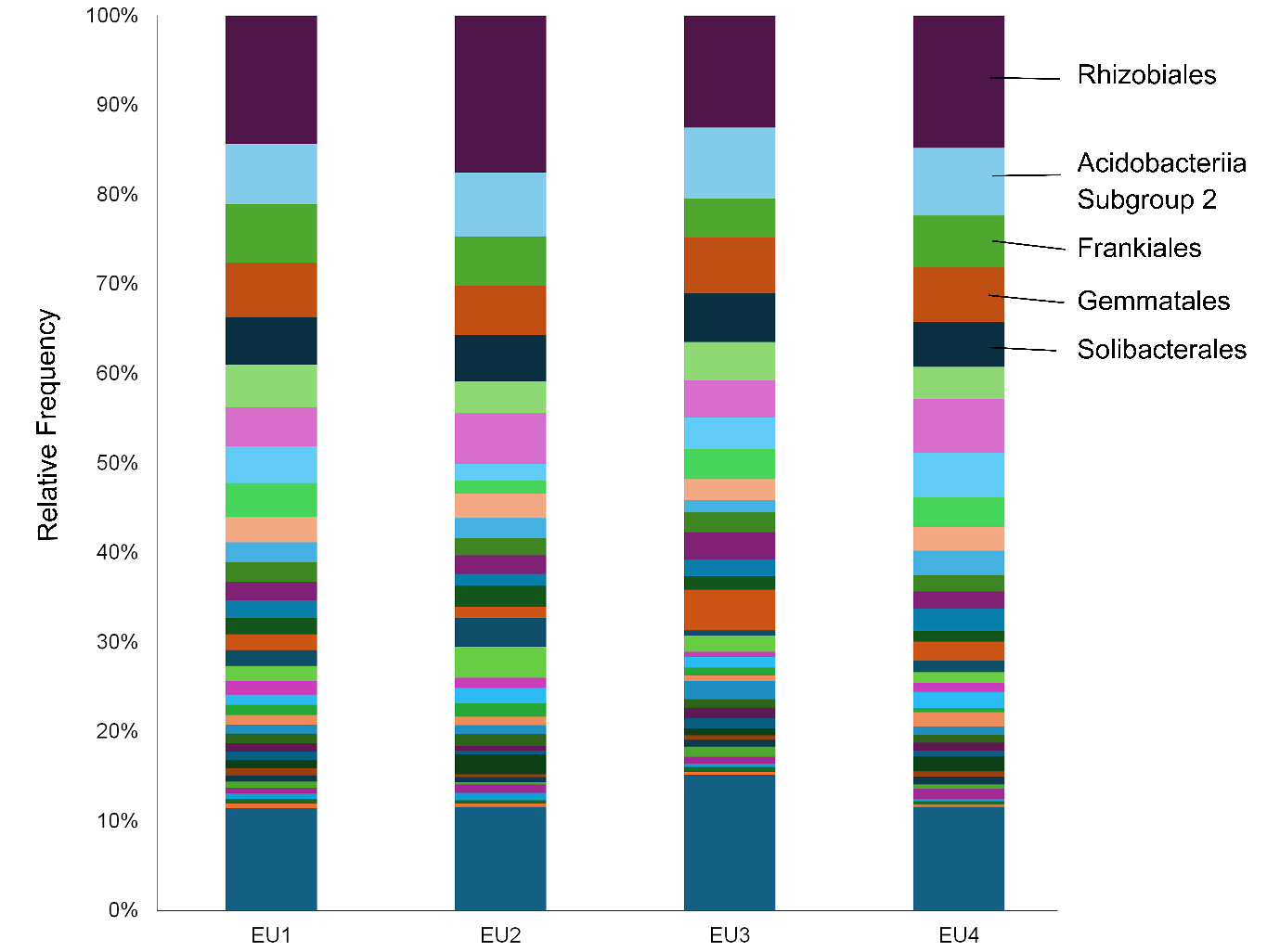


**FIGURE S2**. The most abundant bacterial order associated with *E. saligna* in Magoebaskloof, Limpopo Province, South Africa.


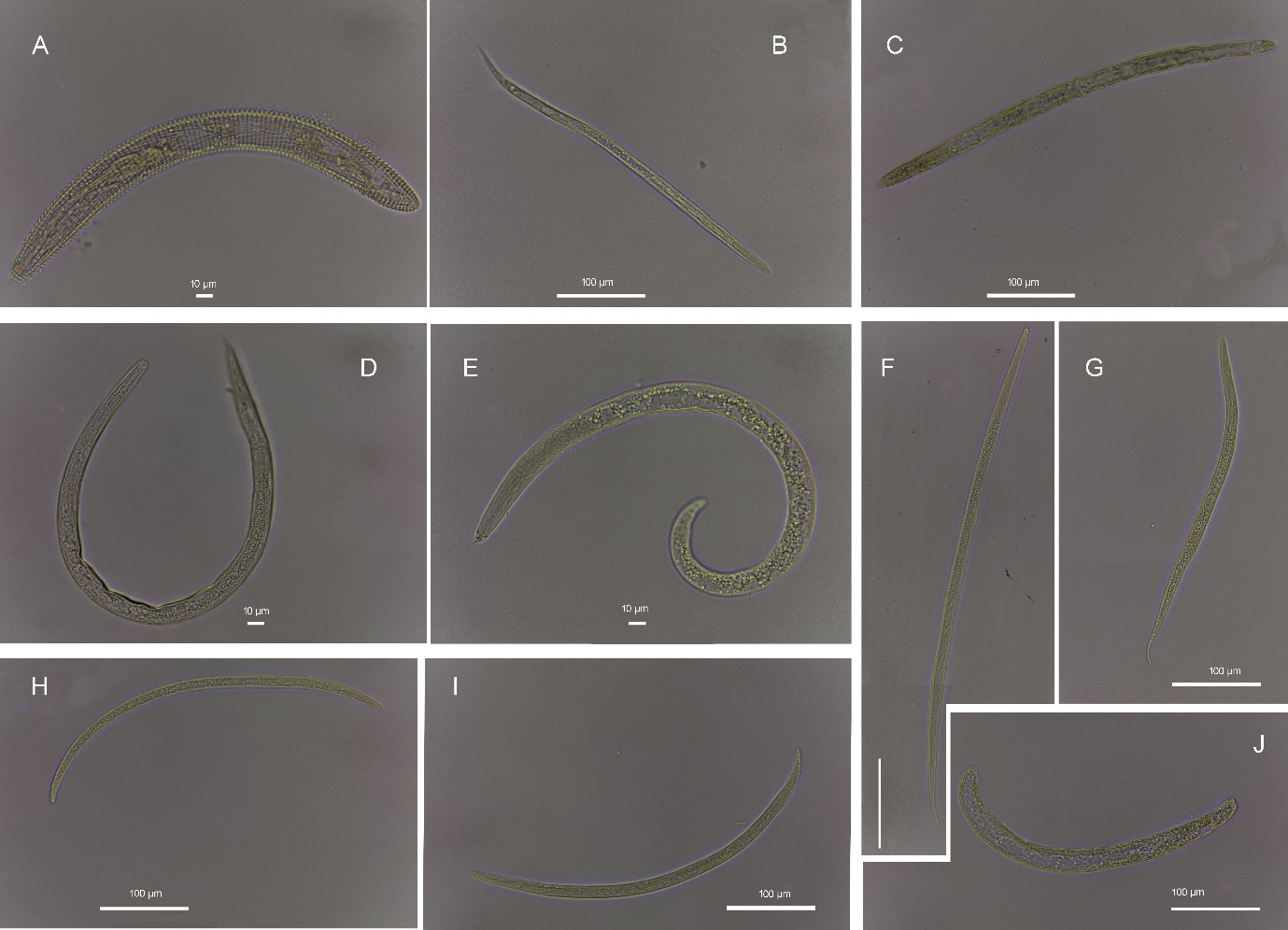


**FIGURE S3.** A: *Xenocriconemella*; B: *Meloidogyne*; C: *Paratrichodorus*; D: *Paratylenchus*; E: *Rotylenchulus*; F: *Ditylenchus*; G: *Tylenchus*; H: *Aphelenchus*; I: *Aphelenchoides*; J: *Tylolaimorphus*.


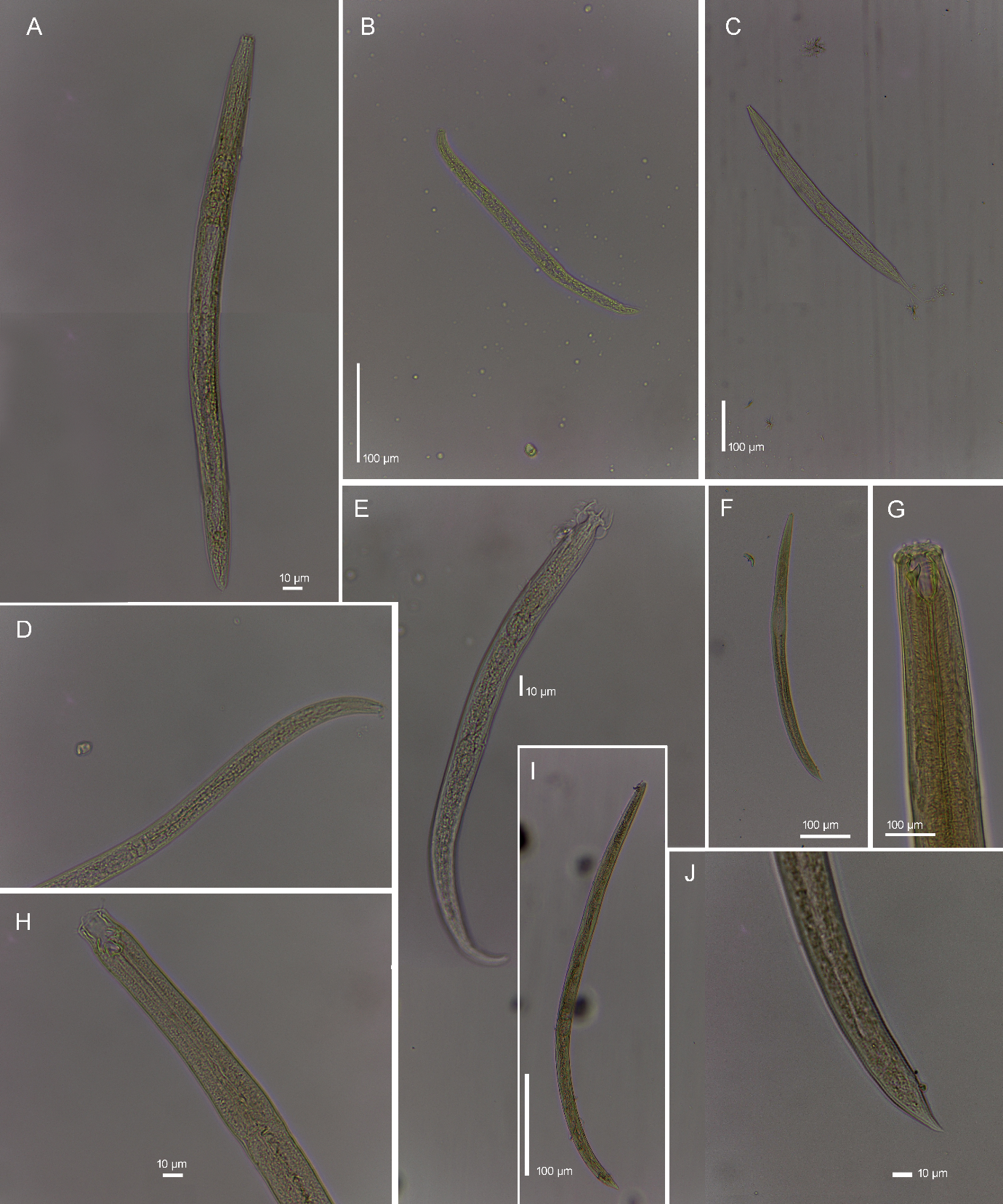


**FIGURE S4**. A: *Acrobeloides*; B: *Cervidellus*; C: *Mesorhabditis*; D: *Prismatolaimus*; E: *Wilsonema*; F, J: *Zeldia*; G: *Mylonchulus*; H: *Butlerius*; I: *Tylencholaimus*.
